# Multicenter Real-World Observational Study of Pargeverine/Lysine Clonixinate in Acute Visceral Colicky Pain

**DOI:** 10.3390/medicina62040706

**Published:** 2026-04-07

**Authors:** Gerardo E. Espinosa-Estrada, Samuel Sevilla-Fuentes, Brandon Bautista-Becerril, Ramcés Falfán-Valencia, Luis Ángel Mendoza-Vargas, José Francisco Araiza-Rodríguez, Pedro Moreno-Chavez

**Affiliations:** 1 Kuatro Gastroenterology Medical Specialties Tower, Metepec 52177, Mexico; 2 Hospital General de Zona 1 “Emilio Varela Luján”, Zacatecas 98000, Mexico; 3 Laboratorio de Neumogenómica, Instituto Nacional de Enfermedades Respiratorias Ismael Cosío Villegas, Mexico City 14080, Mexico; rfalfanv@iner.gob.mx; 4 Hospital General de Zacatecas “Luz González Cosío”, Zacatecas 98160, Mexico; 5 Hospital “Grupo Ángeles, México”, General Surgery, Mexico City 10700, Mexico

**Keywords:** colicky abdominal pain, pargeverine, lysine clonixinate, abdominal pain, antispasmodic therapy, GI symptoms, pharmacological treatments, functional gastrointestinal disorders

## Abstract

*Background and objectives:* Acute colicky abdominal pain, involving visceral spasms and inflammatory mechanisms, is a common complaint in everyday medical practice. Despite its widespread use, clinical evidence on the fixed-dose combination of pargeverine 10 mg plus lysine clonixinate 125 mg remains limited. The objective of this study was to describe real-world clinical outcomes, safety, and patient-reported experience associated with this combination in routine practice. *Materials and methods:* This multicenter, observational, non-comparative, real-world study included adult patients with acute colicky abdominal pain of gastrointestinal, urological, or gynecological origin. Pain intensity was assessed using a visual analog scale (VAS) at baseline and at 5 ± 3 days. Secondary outcomes included time to pain relief, satisfaction with treatment, and safety. Associations between pain reduction and patient-reported outcomes were explored. *Results:* A total of 202 patients were analyzed. Significant pain reduction was observed across all etiologies (*p* < 0.001), with median VAS reductions ranging from 6 to 9 points (approximately 70% to 90% from baseline). More than 95% of patients reported early improvement, and sustained relief was maintained in more than 96% throughout the study period, regardless of the pain’s origin. Adverse events were infrequent (3%), mild in intensity (primarily transient gastrointestinal symptoms), and did not lead to treatment discontinuation. *Conclusions:* In real-world clinical practice, the fixed-dose combination of pargeverine and lysine clonixinate was associated with early and sustained, clinically meaningful pain reduction across multiple forms of acute visceral colicky pain, with a favorable safety and tolerability profile, supporting its relevance as a short-term therapeutic option in mild to severe acute pain.

## 1. Introduction

Abdominal pain is a common gastrointestinal symptom characterized by intermittent spasmodic contractions that produce acute episodes of discomfort that intensify, peak, and then subside without completely disappearing. It is usually caused by distension, inflammation, or obstruction of hollow organs and, although often benign, it can also indicate clinically significant underlying conditions that require timely and thorough evaluation [1]. This type of pain remains one of the most common reasons for medical consultation, accounting for up to 40% of outpatient visits and approximately 5% to 10% of emergency department visits [2,3].

This type of pain is also frequently associated with a broad spectrum of gastrointestinal, urinary, and gynecological conditions [4,5]. Its treatment usually includes the use of nonsteroidal anti-inflammatory drugs (NSAIDs) and antispasmodic agents aimed at reducing both the nociceptive and spasmodic components of pain [6,7,8,9]. In several Latin American countries, one of the most commonly used combinations for this purpose is pargeverine combined with lysine clonixinate [9,10,11,12].

Pargeverine is a well-established antispasmodic indicated for the treatment of colicky visceral pain [1,13,14]. It combines antimuscarinic and calcium channel-blocking properties, promoting smooth muscle relaxation in gastrointestinal and genitourinary tissues [1,15]. This dual mechanism underlies its antispasmodic effect and may contribute to a favorable tolerability profile compared with single-mechanism agents [1,13,15,16,17].

Lysine clonixinate is a nonsteroidal anti-inflammatory drug (NSAID) with analgesic and anti-inflammatory properties [18]. It has demonstrated favorable tolerability, with low evidence of hepatic or renal toxicity and a low incidence of gastrointestinal adverse events reported during extensive clinical use [19,20]. Additionally, it does not alter coagulation parameters and exhibits preferential cyclooxygenase-2 (COX-2) inhibition, which may reduce the risk of adverse effects commonly associated with non-selective NSAIDs [19,21,22].

Although this combination has been used for decades in the management of colicky abdominal pain, real-world clinical data remain limited [6]. This gap restricts a comprehensive understanding of its effectiveness, tolerability, and therapeutic value across the diverse etiologies associated with spasmodic abdominal pain.

This study aims to present a multicenter real-world analysis of the clinical experience with this combination, describing its therapeutic response, safety profile, and clinical relevance in the management of colicky abdominal pain.

## 2. Materials and Methods

### 2.1. Study Design and Population

This multicenter, observational, non-comparative real-world study evaluated the clinical experience with the fixed-dose combination of pargeverine and lysine clonixinate in adult patients presenting with acute colicky abdominal pain. The study was conducted without a control or comparator arm, as it was designed to reflect routine clinical practice without intervention beyond standard care. Therefore, it was conducted in several outpatient centers with the participation of specialists in gastroenterology, infectious diseases, rheumatology, and pain management. All evaluations were performed in accordance with standard clinical practice, without influencing the diagnostic pathways or therapeutic decisions made by the treating physicians. As this was a multicenter study, medical centers in Mexico were included from June to December 2025, including Zacatecas (n = 73), Aguascalientes (n = 48), the State of Mexico (n = 43), and Mexico City (n = 38).

Eligible participants were adults aged 18 years or older presenting with colicky abdominal pain of ≤48 h duration, regardless of intensity. Diagnostic classifications across participating centers followed established clinical criteria and guideline-based definitions applicable to each condition (e.g., Rome IV for irritable bowel syndrome [23]), ensuring consistency in case identification, infectious gastroenteritis, and functional colitis; uncomplicated biliary colic; uncomplicated upper or lower urinary tract infections with a colicky pain pattern; uncomplicated renal colic; and primary dysmenorrhea. Additionally, patients with other non-emergent organic conditions capable of producing colicky abdominal pain were eligible for inclusion. Only first-episode or new-onset cases were enrolled, and patients were required not to have used analgesics or antispasmodics in the preceding 12 h.

Patients were excluded if they presented alarm features such as fever, persistent vomiting, gastrointestinal bleeding, jaundice, unintentional weight loss, severe dehydration, or a palpable abdominal mass [2]. Additional exclusion criteria included severe chronic conditions such as advanced liver disease, decompensated renal impairment, significant cardiovascular disease, or active cancer. Pregnant or breastfeeding women, individuals with known hypersensitivity to pargeverine or lysine clonixinate, and patients with suspected emergency-related gynecological or abdominal etiologies were also excluded. Further exclusions applied to individuals who had participated in another clinical study within the preceding 30 days or those who, for any reason, were unable to provide informed consent or reliably complete the study assessments.

Concomitant therapies deemed clinically necessary (e.g., antibiotics for infectious etiologies) were permitted under routine clinical practice. Rescue analgesics were also permitted at the treating physician’s discretion. Their use was recorded, and patients were not excluded from the analysis if isolated rescue doses were administered.

Given its observational, non-interventional design and minimal risk, all patients provided informed consent for diagnosis and treatment as part of routine clinical care at each participating institution. In addition, written informed consent was obtained from all participants for the collection and analysis of anonymized patient-reported survey data.

An initial reference sample size was calculated using a standard formula for proportions in large populations, assuming a 95% confidence level, an expected proportion of 0.5, and a margin of error of 10%, yielding a minimum of approximately 96 participants [24].

To enhance the robustness of descriptive estimates and account for potential losses or incomplete data, patient recruitment was continued using a consecutive sampling strategy during routine clinical consultations. As patient flow exceeded initial projections, 202 eligible patients were ultimately included during the predefined recruitment period. This approach allowed for a more comprehensive representation of clinical practice while maintaining the study’s descriptive and exploratory objectives.

### 2.2. Study Procedures and Outcome Assessment

Once eligibility was confirmed, baseline demographic and clinical data were collected during the initial consultation. All patients received standard medical management with the fixed-dose combination (Plidán Compuesto^®^, Weser Pharma; CDMX, México) containing pargeverine hydrochloride 10 mg and lysine clonixinate 125 mg per tablet, administered as two tablets every eight hours (total daily dose: 60 mg of pargeverine and 750 mg of lysine clonixinate).

Treatment duration was determined according to routine clinical practice and the underlying condition, with an expected course of approximately 5 days. Follow-up assessments were conducted within the predefined window (5 ± 3 days), with the vast majority occurring near day 5 and without significant differences across centers. During both visits, an external evaluator independent from the treating medical team administered a structured questionnaire to minimize assessment bias. This instrument evaluated the evolution of colicky abdominal pain using a visual analogue scale (VAS), time to onset of symptomatic relief, progression or resolution of accompanying symptoms, presence of adverse events, global perception of therapeutic effectiveness and treatment satisfaction. All data were anonymized before analysis.

A reduction of ≥5 points on the 10-point VAS was selected to represent a clinically meaningful improvement, based on previously established thresholds in clinical pain research defining substantial response as approximately ≥50% reduction in pain intensity [25,26].

Loss to follow-up was defined as failure to attend the scheduled follow-up visit, completion of questionnaires < 70%, initiation of additional analgesic or antispasmodic therapy before the follow-up assessment, or voluntary withdrawal from the study.

### 2.3. Statistical Analysis

All statistical analyses were performed using SPSS version 31.0 and RStudio 4.5.2 (libraries: ggplot2, corrplot, tidyverse, and Hmisc). Quantitative variables were assessed for distribution and summarized using means, medians, and standard deviations, as appropriate. Qualitative variables were reported as absolute and relative frequencies. Normality was evaluated using the Kolmogorov–Smirnov test and distribution parameters.

To explore associations among clinical characteristics, patient-reported outcomes, and symptomatic improvement, the Mann–Whitney U test was used for between-group comparisons, and Spearman’s rank correlation coefficient was used to assess relationships among continuous variables. Changes in pain intensity between baseline and day 5 were analyzed using the paired Wilcoxon signed-rank test. Effect size (r) was calculated as Z/√N and interpreted according to Cohen’s conventional thresholds (0.10 = small, 0.30 = medium, 0.50 = large effect).

Analyses were performed for the overall population and stratified by pain etiology. Missing or incomplete questionnaires were excluded from the corresponding analyses following a per-protocol approach. A two-sided *p*-value < 0.05 was considered statistically significant.

## 3. Results

A total of 220 patients were included; however, 18 were considered to have failed to return for follow-up consultations, resulting in 202 patients from multiple outpatient care centers. The demographic and clinical characteristics stratified by pain etiology are presented in Table 1.

Baseline pain intensity was high in all etiological groups, with mean VAS scores indicating moderate to severe pain and slightly higher baseline values in the gynecological group. After 5 ± 3 days of treatment, a marked reduction in pain intensity was observed across all gastroenterological, gynecological, and urological etiologies. Clinically significant improvements were reflected in substantial median ΔVAS values in all groups, with reductions of 7 points in gastroenterological pain, 6 points in urological pain, and 7.5 points in gynecological pain.

### 3.1. Perception of Pain Relief and Early Clinical Response

**Figure 1:**
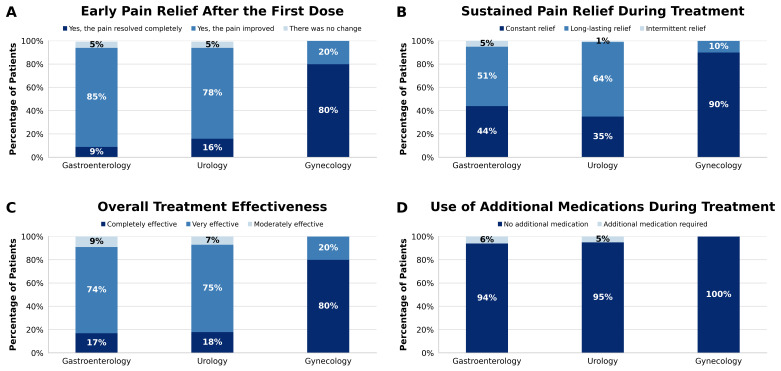
Patient-reported treatment response and perceived effectiveness across pain etiologies. (**A**): Early pain relief after the first dose. (**B**): Sustained pain relief during treatment. (**C**): Overall treatment effectiveness. (**D**): Use of additional medications during treatment.

Patients’ reported pain relief assessed early clinical response after the first dose. As shown in Figure 1A, most patients reported early improvement in pain, either partial or complete, shortly after starting treatment. Overall, more than 95% of patients across all etiological groups reported early pain improvement, including 95% with gastrointestinal pain, 94.6% with urological pain, and 100% with gynecological pain, indicating a clinically noticeable onset of action that was consistent across all etiologies evaluated.

To determine whether this initial improvement was sustained over time, the persistence of pain relief during the treatment period was subsequently analyzed. Overall, 96% of patients reported sustained pain relief throughout treatment. As illustrated in Figure 1B, 95% of patients with gastrointestinal pain, 98% with urological pain, and 100% with gynecological pain reported sustained or complete relief, with only a small minority reporting very intermittent relief. These results suggest that, in most cases, the early clinical response was maintained throughout the treatment period.

The overall patient-reported effectiveness ratings, supported by VAS evolution, remained high across all etiologies. In the overall cohort, the treatment was rated as very effective or completely effective by more than 92% of patients. As shown in Figure 1C, 91% of patients in the gastroenterology group, 92.7% in the urology group, and 100% in the gynecology group reported high perceived effectiveness. Reports of no effectiveness were rare (0.5% in the gastroenterology group), and no patients in the gynecological or urological groups rated the treatment as ineffective.

Finally, the use of additional analgesic medication during treatment was infrequent (Figure 1D). Most patients did not require rescue therapy, including 94% of those with gastroenterological pain, 95% of those with urological pain, and 100% of those with gynecological pain. When additional medications were used, they consisted of isolated doses, as needed, of commonly prescribed analgesics such as acetaminophen, ketorolac, or tramadol, without discontinuing treatment.

**Figure 2:**
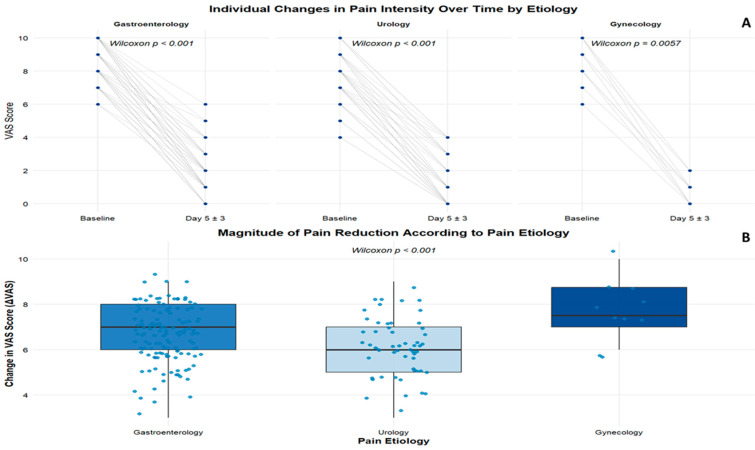
Reduction in pain intensity from baseline to day 5 ± 3 according to pain etiology. (**A**): Individual Pain Trajectories from Baseline to Day 5 ± 3. (**B**): Magnitude of Pain According to Pain Etiology. VAS: Visual Analogue Scale.

To further address the study objectives, Figure 2 illustrates individual pain trajectories showing a marked decrease in visual analog scale (VAS) scores from baseline to day 5 ± 3 after the start of treatment. In the overall cohort, this reduction was statistically significant, as demonstrated by a paired Wilcoxon signed-rank test (V = 20,503; *p* < 0.001), with a large effect size (r = 0.87), indicating a substantial and consistent reduction in reported pain intensity during follow-up.

When the analysis was stratified by pain etiology, the reduction in VAS scores remained statistically significant in all subgroups. Patients with gastroenterological pain showed a marked reduction in pain intensity from baseline to day 5 ± 3 (n = 137; Wilcoxon signed-rank test, V = 9453; *p* < 0.001), with a large effect size (r = 0.87). Similar results were observed in patients with urological pain (n = 55; *p* < 0.001; r = 0.88). A statistically significant reduction was also observed in the gynecological subgroup (n = 10; *p* = 0.006; r = 0.87); however, given the small sample size, these results should be interpreted with caution. Overall, effect sizes were consistently large across all etiologies, supporting a robust within-group improvement in reported pain intensity.

Figure 2B shows the distribution of pain reduction (ΔVAS) according to pain etiology. A statistically significant difference in the magnitude of pain reduction was observed between etiological categories (Kruskal–Wallis χ^2^ = 14.75, df = 2, *p* < 0.001). Post hoc comparisons performed with Dunn’s test with Holm adjustment demonstrated significant differences between all etiological pairs, with the greatest mean reduction observed in patients with gynecological pain, followed by those with gastroenterological and urological pain. It is important to note that, despite these statistical differences, all groups showed clinically significant improvements, with high effect sizes and mean ΔVAS values greater than 6 points.

These findings are further detailed in Table 2, which summarizes median VAS values at baseline and follow-up, as well as absolute and relative pain reductions by pain etiology and clinical diagnosis. At the etiological level, patients with gastroenterological pain experienced a marked decrease in pain intensity, with median ΔVAS values consistently around 7 points across most diagnostic categories, corresponding to relative reductions of approximately 60% to 90% from baseline. Notably, in several gastroenterological conditions, baseline pain intensity reached the upper end of the VAS, indicating that even partial reductions represented a clinically meaningful decrease in pain burden.

A similarly consistent pattern was observed in the urological group, where median absolute pain reductions were typically 6–7 VAS points, with relative improvements most frequently ranging from approximately 60% to 100%, depending on the underlying diagnosis. In patients with urinary tract infection, complete or near-complete pain relief was frequently reported, whereas slightly lower relative reductions were observed in conditions such as renal lithiasis.

In the gynecological subgroup, although the number of patients was smaller, the magnitude of pain reduction remained notable. Median decreases of 7 to 8 VAS points were observed, with relative reductions generally approaching or exceeding 90%, particularly in patients with primary dysmenorrhea. Overall, despite differences in baseline pain severity and clinical diagnosis, all etiological groups demonstrated substantial and clinically meaningful improvements in pain intensity during the short-term follow-up period.

### 3.2. Safety and Tolerability Profile

**Figure 3:**
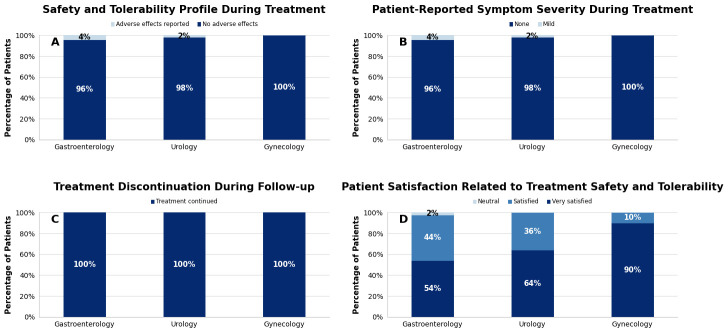
Safety, tolerability, and patient-reported experience across pain etiologies. (**A**): Occurrence of adverse events during treatment. (**B**): Severity of reported adverse events. (**C**): Treatment discontinuation rates. (**D**): Overall patient experience after treatment completion.

Overall, the treatment demonstrated a favorable safety and tolerability profile across all evaluated etiological groups. As illustrated in Figure 3A, the proportion of patients reporting additional symptoms or adverse events during treatment was low. Adverse events were observed in 4% of patients in the gastroenterological group and 2% in the urological group, whereas none were reported in the gynecological group. Overall, adverse events were reported in 3% of the total study population.

Regarding symptom severity, Figure 3B shows that all reported adverse events were mild, defined as symptoms that did not interfere with patients’ daily activities. Importantly, no moderate, severe, or very severe symptoms were observed in any etiological category. The reported events mainly consisted of transient and self-limiting manifestations, such as nausea, vomiting, or dry mouth. Given the observational nature of the study and the absence of a control group, these mild symptoms cannot be conclusively attributed to the treatment.

As shown in Figure 3C, no patients discontinued treatment due to adverse events or newly emerging symptoms during follow-up. In addition, patient-reported experience following treatment completion was predominantly favorable. As illustrated in Figure 3D, 98% of patients in the gastroenterological group and 100% of patients in both the urological and gynecological groups rated their satisfaction as “satisfied” or “very satisfied” about the safety and tolerability of the treatment.

### 3.3. Patient-Reported Treatment Experience

**Figure 4:**
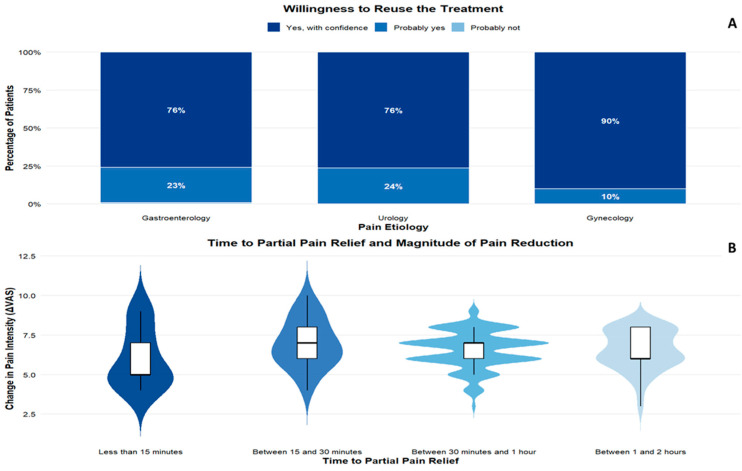
Patient-reported onset of pain relief and willingness to reuse the treatment. (**A**): Willingness to reuse the treatment by etiology. (**B**): Association between time to partial pain relief and magnitude of pain reduction.

In Figure 4A, we observe that a large majority of participants expressed their intention to use the medication again, selecting “yes, with confidence” or “probably yes.” This response pattern was observed in more than 99% of the total study population. The willingness to use the treatment again was uniformly positive in the gynecological and urological colic pain groups, while only 0.7% of gastroenterological patients responded: “probably not.” Overall, these findings reflect a favorable perception of the treatment by patients after the resolution or improvement of the acute pain episode.

The relationship between the perceived onset of analgesic relief and the magnitude of pain reduction is explored in Figure 4B. Differences in pain reduction were observed according to the time elapsed until perceived partial relief (Kruskal–Wallis test: χ^2^ = 14.75; df = 3; *p* = 0.002). Patients who reported earlier pain relief tended to show greater mean reductions in VAS scores at the end of treatment.

However, clinically significant improvements were observed across all time categories, including patients who reported initial relief within the first 15 min or within the first 2 h after treatment initiation. These findings indicate that, while earlier perception of the onset of analgesic effect is associated with greater overall pain reduction, significant clinical benefit is maintained over a wide time interval, reflecting expected interindividual variability in response.

An exploratory logistic regression analysis was performed to identify variables associated with clinically meaningful pain reduction (ΔVAS ≥ 5). Each 1-point increase in baseline VAS score was associated with an almost twofold increase in the odds of achieving clinically meaningful pain reduction after adjustment for age and sex; baseline VAS score remained independently associated with clinically meaningful pain reduction (OR = 1.96; 95% CI: 1.25–3.16; *p* = 0.004). This finding suggests that patients with higher baseline pain intensity were more likely to experience a significant therapeutic response.

## 4. Discussion

This observational study describes the real-world clinical experience with the fixed-dose combination of pargeverine and lysine clonixinate in patients presenting with colicky abdominal pain of different etiologies. Overall, the results show a rapid and sustained reduction in pain intensity during the treatment period, with good tolerability and a generally favorable experience. It is important to note that these results were observed in gastrointestinal, urological, and gynecological pain conditions. Taken together, these findings describe consistent patterns of pain reduction across different forms of visceral spastic pain, regardless of the underlying cause.

Although multiple etiologies were included, all share a common pathophysiological component of visceral smooth muscle spasm and inflammatory-mediated nociception, which supports the rationale for pooled analyses. Nevertheless, differences in baseline characteristics and underlying conditions should be considered when interpreting subgroup-specific results.

Physiological gastrointestinal motility depends on the coordinated interactions among smooth muscle cells (SMCs), neural, hormonal, and inflammatory responses [27,28]. In visceral colic, this rhythmic pacemaker activity becomes disrupted by enteric neuronal hyperstimulation. Sustained acetylcholine release leads to muscarinic receptor overactivation and increased intracellular calcium availability from both extracellular influx and internal stores [29,30]. The resulting calcium overload amplifies contractile force and disrupts smooth muscle coupling, shifting coordinated peristalsis toward disorganized, high-amplitude spasmodic contractions that underlie acute colicky pain.

Within this framework, pargeverine directly targets the key pathways involved in spastic contraction. Through dual muscarinic antagonism and calcium channel modulation, it interferes with both cholinergic overstimulation and calcium overload in smooth muscle cells [1,13]. This dual mechanism provides a coherent pharmacological rationale for its antispasmodic activity and may contribute to a favorable tolerability profile compared with single-mechanism agents [16,17].

The pharmacological relevance of pargeverine is supported by experimental studies in human colon tissue demonstrating significant attenuation of carbachol-induced and neurogenic contractions, confirming its direct antispasmodic efficacy at the level of smooth muscle [16].

Notably, pargeverine also reduced the occurrence of disorganized “outlier” contractions associated with motor instability, suggesting selective modulation of excitatory pathways involved in visceral spasm while preserving coordinated neuromuscular signaling [16].

Muscarinic acetylcholine receptors (M1–M5) mediate cholinergic signaling across visceral tissues involved in colicky pain syndromes [28]. Among these, M2 and M3 receptors play a central role in gastrointestinal smooth muscle contraction by increasing intracellular calcium availability following acetylcholine release [31]. Activation of these receptors triggers intracellular signaling pathways that increase calcium availability, promoting smooth muscle contraction and contributing to the pathophysiology of visceral spasm and colic pain.

Within this framework, receptor affinity represents a key determinant of both clinical efficacy and tolerability. Experimental data indicate preferential interaction of pargeverine with M2–M3 muscarinic receptor subtypes predominantly involved in intestinal smooth muscle contraction, while demonstrating comparatively lower affinity for central muscarinic receptors. This selective pharmacological profile supports potent peripheral antispasmodic activity with limited systemic involvement and could translate into a potent antispasmodic effect on smooth muscle, with up to a 432-fold greater relative potency than butylscopolamine in carbachol-induced contraction models, as calculated based on previously reported experimental data [16].

This functional selectivity may contribute to effective spasm control while maintaining a favorable safety profile.

Following administration of the combination therapy, the vast majority of patients across all etiologies reported early improvement. Importantly, pain relief was maintained throughout the treatment period in more than 96% of patients, particularly among those who responded early. Initial relief was noted within 15 min; notably, the most substantial absolute VAS reductions were consistently achieved even in patients requiring a broader window for onset.

These observations are consistent with previous clinical studies evaluating pargeverine in the treatment of visceral colic. Mezzotero et al. reported significant pain reduction within 30 min after a single oral dose in patients with mild to moderate biliary, intestinal, renal, and gynecological colicky pain [14]. Similarly, in the present cohort, clinically meaningful reductions were documented even among patients with severe baseline pain, reinforcing the robustness of the analgesic response across etiologies.

These findings align with prior controlled studies of acute visceral pain. De los Santos et al. evaluated pargeverine in 350 patients with moderate to severe biliary colic and reported progressive reductions in pain intensity compared with placebo across doses. At 120 min, complete pain relief was achieved in 60% of patients receiving pargeverine, compared with 28% in the placebo group [32], supporting its clinical effectiveness in acute colicky syndromes.

Functional high affinity for the M2 and M3 muscarinic receptor subtypes may contribute to effective smooth muscle relaxation while limiting systemic involvement. In this regard, our findings showed that this tolerability profile was also reflected in the low incidence of adverse events (<3%), all of which were mild and transient, without clinically relevant cardiovascular or central nervous system effects [1,16].

This favorable tolerability profile aligns with prior clinical evidence. Rzetelna et al. demonstrated that the combination was well-tolerated, with no significant alterations in vital signs (including blood pressure, respiratory rate, or heart rate) across treatment groups, further reinforcing its systemic safety [1]. Similarly, De los Santos et al. observed good tolerability across all evaluated doses (10–30 mg), with no discontinuations due to adverse events. Dry mouth was the most frequently reported event at higher doses, without clinical relevance or need for treatment interruption [32].

The analgesic effects of lysine clonixinate (LC) in visceral pain have also been documented. In a randomized, double-blind crossover trial in primary dysmenorrhea, LC significantly reduced abdominal colicky pain (from 2.9 ± 0.7 to 0.66 ± 0.4; *p* < 0.0001), without significant adverse effects [10].

In addition, Álvarez et al. evaluated LC 125 mg combined with pargeverine 10 mg in acute abdominal pain of various etiologies, reporting a 43% reduction in VAS scores within the first hour and pain levels below 1 by 4 h, alongside high satisfaction among physicians (81%) and patients (78%) [6].

In our study, the combination of LC and pargeverine was associated with rapid and sustained pain relief, with mean VAS reductions of 6–8 points across etiological groups, including patients presenting with severe cramping pain. Prior controlled data support these observations. In a randomized, double-blind, crossover trial in 125 patients with primary dysmenorrhea, LC 125 mg plus pargeverine 10 mg was compared with paracetamol plus hyoscine N-butylbromide and placebo [9]. Although both active treatments reduced pain intensity versus placebo, only the LC–pargeverine combination maintained statistically superior analgesic effects on days three and four, suggesting more sustained pain control in acute colicky conditions [9].

**Figure 5:**
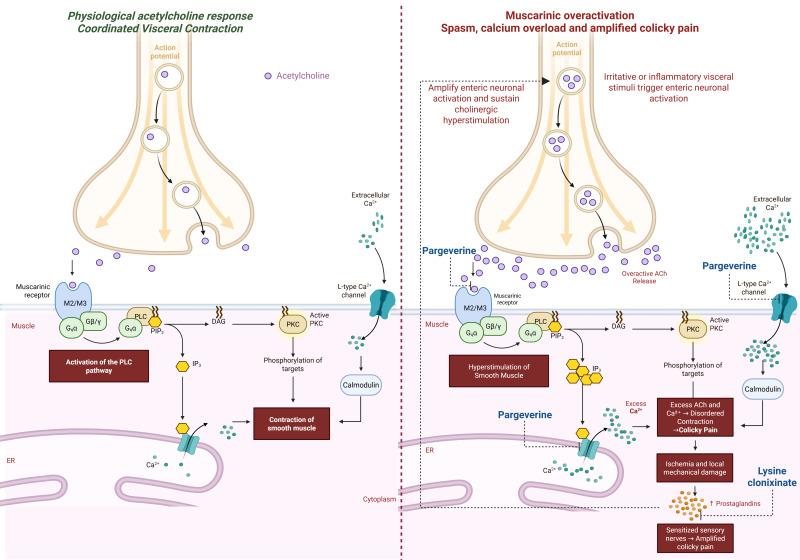
Mechanistic Model of Pargeverine and Lysine Clonixinate in Visceral Colicky Pain. Transition from physiological acetylcholine-mediated contraction (**left**) to muscarinic overactivation, calcium overload, and prostaglandin-driven neuronal sensitization (**right**). Pargeverine exerts dual action through muscarinic receptor antagonism and calcium channel blockade at sarcoplasmic and membrane levels. Lysine clonixinate reduces prostaglandin-mediated sensitization and inflammatory amplification. ACh = acetylcholine; IP_3_ = inositol trisphosphate; DAG = diacylglycerol; PKC = protein kinase C; ER = endoplasmic reticulum; Ca^2+^ = calcium. Created in BioRender. Bautista-Becerril, B. & Falfán-Valencia, R. (2026).

These findings are consistent with the hypothesis that dual targeting of muscarinic calcium overload and nociceptive sensitization may contribute to pain reduction in colicky syndromes. Based on this interpretation, we propose a mechanistic model summarizing the pathophysiological cascade and the site of action of both pargeverine and lysine clonixinate (Figure 5).

Taken together, these findings suggest that LC, particularly when combined with musculotropic agents, may provide rapid and sustained relief of acute gynecological pain. In our cohort, 57.7% of patients presented with gastrointestinal pain, most commonly associated with irritable bowel syndrome (IBS), including diarrhea-predominant, constipation-predominant, and mixed subtypes. Although the role of NSAID-containing combinations in IBS remains debated, 93.7% of patients reported early symptom relief after the first dose, and 96.2% described sustained improvement during the treatment period.

Similar observations have been reported in IBS populations. Pulpeiro et al., in a prospective placebo-controlled study of 75 patients, documented significant reductions in abdominal pain, cramping frequency, and postprandial bloating in the pargeverine group compared with placebo, with improvements evident from the first week and no treatment discontinuations due to adverse events [17].

In summary, our data indicate that the pargeverine/lysine clonixinate combination provides meaningful symptomatic relief during acute episodes of visceral pain. While regional guidelines prioritize antispasmodic monotherapy for the long-term management of IBS and specifically caution against the routine use of NSAID-containing combinations, our observations focus on a distinct clinical window [33]. These findings suggest that such combinations may serve as a short-term therapeutic option for acute exacerbations where rapid symptom control is prioritized, rather than as a maintenance therapy for the underlying syndrome.

A strong point of this study is the provision of clinically relevant real-world data on pain reduction and tolerability associated with the combination of pargeverine and lysine clonixinate in the treatment of acute visceral pain of gastrointestinal, urological, and gynecological origin. Although there are a few prior reports, many are based on preclinical studies or non-human models, underscoring the need for contemporary clinical evidence from real-world practice settings. In this context, the present study provides original and relevant clinical information, contributing to expanding current knowledge in an area that remains relatively unexplored.

Several limitations must also be acknowledged. The number of patients with gynecological pain, particularly those with primary dysmenorrhea, was relatively small, limiting the strength of subgroup-specific conclusions. In addition, the observational design and absence of a comparator group should be considered when interpreting these findings. As in most real-world studies of acute conditions, factors such as the natural course of symptoms and concomitant management may have influenced the magnitude of pain reduction observed.

Additionally, early pain relief was assessed based on patients’ perceptions at follow-up, in keeping with the study’s real-world design. The relatively short follow-up period reflects the acute nature of the treated conditions and routine clinical practice. While no serious safety signals were identified during the observation window, longer follow-up would be needed to evaluate rare or delayed adverse events comprehensively.

Furthermore, as an initial exploratory phase, the study reflects the experience of a limited number of participating centers. This work represents the first step in a broader research program, with plans to expand patient selection, increase the number of participating investigators, and strengthen subgroup analyses in future phases.

## 5. Conclusions

This multicenter real-world study describes clinically meaningful pain reduction and tolerability associated with the fixed-dose combination of pargeverine and lysine clonixinate in acute visceral colicky pain. The treatment was associated with rapid and sustained pain relief across gastrointestinal, urological, and gynecological etiologies, with good tolerability and favorable patient-reported outcomes. Together, these findings reinforce the clinical relevance of this combination as a therapeutic option for acute episodes of visceral pain.

## Figures and Tables

**Table 1:** Demographic characteristics and clinical evolution according to the origin of pain.

Variable	Gastroenterological n = 137	Urological n = 55	Gynecological n = 10	*p*
Age, years	44 (34–54)	46 (34.5–55.5)	23.5 (21.5–29.8)	0.01
Sex (n, %)				
Female	78 (56.9%)	43 (78.2%)	10 (100%)	0.01
Male	59 (43.1%)	12 (21.8%)	0 (0%)	
VAS score (0–10)				
Baseline	8 (8–9)	7 (6–8)	8.5 (8–9.7)	<0.001
Day 5 ± 3 of treatment	2 (1–2)	1 (0–2)	1 (0–1)	<0.001
VAS Differential	7 (6–8)	6 (5–7)	7.5 (7–8.75)	<0.001
Final diagnosis (%)	Irritable Bowel Syndrome (57.7%) Infectious colitis (19.7%) Biliary colic (10.2%) Functional gastrointestinal disorders (5.8%) Dyspepsia (2.2%) Diverticulitis (2.2%) Gallbladder lithiasis (2.2%)	Urinary tract infection (67.3%) Pyelonephritis (18.2%) Renal lithiasis (14.5%)	Primary dysmenorrhea (90%) Pelvic inflammatory disease (10%)	

Continuous data are presented as median (interquartile range, IQR) and categorical data as number and frequency in percentage (%). Statistical tests employed for the comparisons: Kruskal–Wallis test and Fisher’s exact test. (VAS): Visual Analog Scale.

**Table 2:** Distribution of pain reduction from baseline to follow-up by etiology and diagnosis.

Etiology	Diagnostic	VAS Baseline	VAS 5 ± 3	ΔVAS	ΔVAS (%)
Gastroenterological	Irritable Bowel Syndrome	8 (8–9)	1 (1–2)	7 (6–7.5)	87.5% (75–90.6%)
	Infectious colitis	8 (7–9)	1 (1–2)	7 (6–8)	85.7% (75–92.5%)
	Biliary colic	9.5 (8.2–10)	3 (3–4)	5.5 (5–7)	64.6% (51.4–70%)
	Functional gastrointestinal disorders	9.5 (9–10)	2 (1–2)	8 (7–8)	84.2% (73.7–88.9%)
	Dyspepsia	8 (7.5–8)	0 (0–0.5)	7 (7–7.5)	100% (93.8–98.5%)
	Diverticulitis	9 (8–9)	3 (2–3)	7 (6–7)	77% (72.2–83.3%)
	Gallbladder lithiasis	9 (8.5–9.5)	4 (3.5–4.5)	6 (4.5–6)	60% (48.8–63.3%)
Urological	Urinary tract infection	7 (6–7)	0 (0–1)	7 (6–7)	100% (85.7–100%)
	Pyelonephritis	9 (8–9)	2.5 (1–3)	7 (6–8)	77.8% (66.7–88.9%)
	Renal lithiasis	8.5 (7.8–10)	3.5 (3–4)	5.5 (4–6)	60% (55.4–63.5%)
Gynecological	Primary dysmenorrhea	8 (8–10)	1 (0–1)	8 (7–9)	90% (88–100%)
	Pelvic inflammatory disease	9 (9–9)	2 (2–2)	7 (7–7)	77.8% (single observation)

Data are presented as median (interquartile range, IQR) and categorical data as number and frequency in percentage (%). (VAS): Visual Analog Scale.

## Data Availability

The data supporting the findings of this study are available from the corresponding author upon reasonable request.
